# Phylogeography of the Subgenus *Drosophila* (Diptera: Drosophilidae): Evolutionary History of Faunal Divergence between the Old and the New Worlds

**DOI:** 10.1371/journal.pone.0160051

**Published:** 2016-07-27

**Authors:** Hiroyuki F. Izumitani, Yohei Kusaka, Shigeyuki Koshikawa, Masanori J. Toda, Toru Katoh

**Affiliations:** 1 Department of Natural History Science, Graduate school of Science, Hokkaido University, Sapporo, Hokkaido, Japan; 2 Department of Biological Sciences, Tokyo Metropolitan University, Hachioji, Tokyo, Japan; 3 The Hakubi Center for Advanced Research and Graduate School of Science, Kyoto University, Kyoto, Kyoto, Japan; 4 Hokkaido University Museum, Hokkaido University, Sapporo, Hokkaido, Japan; 5 Department of Biological Sciences, Faculty of Science, Hokkaido University, Sapporo, Hokkaido, Japan; University of Arkansas, UNITED STATES

## Abstract

The current subgenus *Drosophila* (the traditional *immigrans*-*tripunctata* radiation) includes major elements of temperate drosophilid faunas in the northern hemisphere. Despite previous molecular phylogenetic analyses, the phylogeny of the subgenus *Drosophila* has not fully been resolved: the resulting trees have more or less varied in topology. One possible factor for such ambiguous results is taxon-sampling that has been biased towards New World species in previous studies. In this study, taxon sampling was balanced between Old and New World species, and phylogenetic relationships among 45 ingroup species selected from ten core species groups of the subgenus *Drosophila* were analyzed using nucleotide sequences of three nuclear and two mitochondrial genes. Based on the resulting phylogenetic tree, ancestral distributions and divergence times were estimated for each clade to test Throckmorton’s hypothesis that there was a primary, early-Oligocene disjunction of tropical faunas and a subsequent mid-Miocene disjunction of temperate faunas between the Old and the New Worlds that occurred in parallel in separate lineages of the Drosophilidae. Our results substantially support Throckmorton’s hypothesis of ancestral migrations via the Bering Land Bridge mainly from the Old to the New World, and subsequent vicariant divergence of descendants between the two Worlds occurred in parallel among different lineages of the subgenus *Drosophila*. However, our results also indicate that these events took place multiple times over a wider time range than Throckmorton proposed, from the late Oligocene to the Pliocene.

## Introduction

The genus *Drosophila* is the most speciose and intensively studied species assemblage in the family Drosophilidae, yet the genus has long been recognized as paraphyletic since the 1975 report of Throckmorton [1], and this characterization has been confirmed in all subsequent, family-wide phylogenetic studies [2–6]. In the most recent molecular phylogenetic analysis [6], the genus *Drosophila* was considered paraphyletic with respect to 15 different genera. Together, these lineages constitute the tribe Drosophilini and are divided into two major clades: one comprises the genus *Lordiphosa* and the subgenus *Sophophora* of *Drosophila*, and the other includes 14 genera and four subgenera of *Drosophila*, including the subgenus *Drosophila*. The subgenus *Drosophila* is comprised of two main clades [4–14] which are interspersed with eight genera and two other subgenera of *Drosophila* [6]. One clade of the subgenus *Drosophila* had been referred to as the *virilis* section [15] or the *virilis*-*repleta* radiation [1,16], and the other as the *quinaria* section [15] or the *immigrans*-*tripunctata* radiation [1,16]. To resolve this paraphyly, Yassin [6] revised the phylogenetic classification of the subgenus *Drosophila*: the *virilis*-*repleta* clade (the *virilis* section) was revised as the subgenus *Siphlodora*, while the *immigrans*-*tripunctata* clade (the *quinaria* section) was left in the subgenus *Drosophila*. The revised subgenus *Drosophila* includes 21 species groups [6], of which 15 have been regarded as members of the *immigrans*-*tripunctata* clade [4]. They are widely distributed mainly in the northern hemisphere, with four species groups endemic to the Old World (the *ancora*, *bizonata*, *histrio* and *immigrans* groups), 14 to the New World (the *appendiculata*, *calloptera*, *cardini*, *ecuadoriensis*, *guarani*, *guttifera*, *lutzii*, *macroptera*, *pallidipennis*, *peruensis*, *rubrifrons*, *sticta*, *tripunctata* and *xanthopallescens* groups) and three distributed in both (the *funebris*, *quinaria* and *testacea* groups). Some of them, such as the *funebris*, *guttifera*, *quinaria* and *testacea* groups, represent major elements of temperate drosophilid faunas in the northern hemisphere.

The revised subgenera *Siphlodora* and *Drosophila* show parallel biogeographic patterns, i.e., the disjunctions between the Old and the New Worlds in tropical elements (species groups) and in species of temperate species groups. Throckmorton [1] hypothesized that this overall pattern of faunal disjunction between the Old and the New Worlds had emerged in parallel in five lineages (radiations in his descriptions) of Drosophilidae, i.e., in the *Scaptodrosophila* radiation, the sophophoran radiation, the *virilis*-*repleta* radiation (the revised *Siphlodora*), the *immigrans*-*tripunctata* radiation (the revised *Drosophila*) and the *Hirtodrosophila* radiation. However, this hypothesis was not presented in a testable way based on solid evidence of phylogenetic relationships. To remedy this, multiple DNA sequence-based studies have provided estimates of divergence times and biogeographic analyses of Old World and New World groups in some lineages of the Drosophilidae [17–20].

Phylogenetic relationships within the revised subgenus *Drosophila* or the *immigrans*-*tripunctata* radiation have been analyzed in a number of studies based on molecular data [2,3,5,6,12–14,19–24]. However, the resulting trees have varied in topology, especially with respect to the basal branches, and have not supported the monophyly of some species groups. Two recent molecular phylogeographic analyses failed to fully resolve the relationships in the *immigrans*-*tripunctata* radiation with high confidence [19,20]. With less resolved phylogenetic hypotheses, estimates of divergence times and ancestral distributions can become equivocal, and inferences for evolutionary history are controversial. Limited taxon sampling can contribute to these ambiguous results, and in many cases such sampling has been biased to New World taxa [18].

We gathered species widely from both the New World and the Old World, selecting 22 species from the Old World, 21 from the New World, one from the Australasian region and one cosmopolitan species in an attempt to reduce the taxon sampling bias. We estimated phylogenetic relationships by analyzing the nucleotide sequences of three nuclear and two mitochondrial genes and estimated ancestral distributions and divergence times in order to test Throckmorton’s [1] hypothesis about parallel patterns of Old World-New World divergence in the evolution of Drosophilidae.

## Materials and Methods

### Taxon sampling

A total of 45 species were selected as focal ingroup taxa from ten core species groups (*bizonata*, *cardini*, *funebris*, *guarani*, *guttifera*, *histrio*, *immigrans*, *quinaria*, *testacea* and *tripunctata*) of the revised subgenus *Drosophila* (the *immigrans*-*tripunctata* radiation). As outgroup taxa, *D*. *virilis*, *D*. *hydei* and *Idiomyia grimshawi* were selected from the sister clade of the subgenus *Drosophila* [6,19,20]. DNA was extracted from either live or alcohol-preserved specimens. The specimens of *D*. *innubila*, *D*. *recens*, *D*. *tenebrosa* and *D*. *transversa* were kindly provided by Prof. John Jaenike (University of Rochester). The remaining 41 specimens were obtained from the UC San Diego Drosophila Stock Center (DSSC), the Tokyo Metropolitan University (TMU) or field collections (Table 1). We determined the DNA sequences of alcohol dehydrogenase (*Adh*) for 35 species, glycerol-3-phosphate dehydrogenase (*Gpdh*) for 36 species, mitochondrial 28S ribosomal RNA (*28S*) for 45 species, cytochrome c oxidase subunit I (*COI*) for 45 species, and cytochrome c oxidase subunit II (*COII*) for 45 species. GenBank accession numbers of these sequences, along with those for known sequences used in the analyses, and taxonomic and biogeographic information are given in Table 1.

**Table 1:** List of studied species.

Genus	Subgenus	Species group	Species	Distribution	Source (Stock No.)	*Adh*	*Gpdh*	*COI*	*COII*	*28S*
*Drosophila*	*Drosophila*	*bizonata*	*bizonata*	Palearctic, Oriental, Australasian	Kyoto, Japan	AB932648	AB932684	AB932720	AB932765	AB932810
		*cardini*	*acutilabella*	Nearctic, Neotropical	DSSC (15181–2171.10)	AB932645	AB932681	AB932716	AB932761	AB932806
			*arawakana*	Neotropic	DSSC (15182–2261.03)	AB932647	AB932683	AB932719	AB932764	AB932809
			*cardini*	Nearctic, Neotropical	DSSC (15181–2181.03)	AB932650	AB932686	AB932722	AB932767	AB932812
			*cardinoides*	Neotropic	DSSC (15181–2291.00)	AB932651	AB932687	AB932723	AB932768	AB932813
			*dunni*	Neotropic	DSSC (15182–2291.00)	AB932654	AB932690	AB932726	AB932771	AB932816
			*nigrodunni*	Neotropic	DSSC (15182–2311.00)	AB932663	AB932698	AB932741	AB932786	AB932831
			*parthenogenetica*	Neotropic	DSSC (15181–2221.00)	AB932666	AB932701	AB932745	AB932790	AB932835
			*polymorpha*	Neotropic	DSSC (15181–2231.00)	AB932668	AB932703	AB932747	AB932792	AB932837
			*similis*	Neotropic	DSSC (15182–2321.00)	AB932673	AB932708	AB932753	AB932798	AB932843
		*funebris*	*funebris*	Palearctic	TMU	AB932656	AB932692	AB932729	AB932774	AB932819
			*macrospina*	Nearctic	TMU	AB932661	AB932696	AB932736	AB932781	AB932826
			*multispina*	Palearctic, Oriental	TMU	AB932662	AB932697	AB932737	AB932782	AB932827
		*guarani*	*ornatifrons*	Neotropic	DSSC (15172–2151.00)	AB932657	AB932693	AB932730	AB932775	AB932820
		*guttifera*	*guttifera*	Nearctic	TMU	AB932658	AB261155	AB932731	AB932776	AB932821
		*histrio*	*histrio*	Palearctic, Oriental	Sapporo, Japan	AB932659	AB932694	AB932732	AB932777	AB932822
			*sternopleuralis*	Palearctic	Wakayama, Japan	AB932674	AB932709	AB932754	AB932799	AB932844
		*immigrans*	*albomicans*	Palearctic, Oriental, Australasian	TMU	AB033642	AB261148	AB932717	AB932762	AB932807
			*formosana*	Oriental	TMU	AB261131	AB261143	AB932728	AB932773	AB932818
			*hypocausta*	Oriental, Australasia	TMU	AB261133	AB261151	AB932733	AB932778	AB932823
			*immigrans*	Cosmopolitan	TMU	M97638	AB261142	AB932734	AB932779	AB932824
			*nasuta*	Oriental, Afrotropic, Neotropic	TMU	AB261132	AB261144	AB932738	AB932783	AB932828
			*neohypocausta*	Oriental, Australasia	TMU	AB261134	AB261146	AB932739	AB932784	AB932829
			*neonasuta*	Oriental	TMU	AB261138	AB261151	AB932740	AB932785	AB932830
			*pallidifrons*	Australasia	TMU	AB261136	AB261150	AB932744	AB932789	AB932834
			*ruberrima*	Oriental	TMU	AB932672	AB932707	AB932751	AB932796	AB932841
			*siamana*	Oriental	TMU	AB261135	AB261147	AB932752	AB932797	AB932842
		*quinaria*	*angularis*	Palearctic, Oriental	TMU	AB932646	AB932682	AB932718	AB932763	AB932808
			*brachynephros*	Palearctic, Oriental	Sapporo, Japan	AB932649	AB932685	AB932721	AB932766	AB932811
			*curvispina*	Palearctic	Sapporo, Japan	AB932652	AB932688	AB932724	AB932769	AB932814
			*deflecta*	Nearctic	TMU	AB932653	AB932689	AB932725	AB932770	AB932815
			*falleni*	Nearctic	TMU	AB932655	AB932691	AB932727	AB932772	AB932817
			*innubila*	Nearctic	University of Rochester	AB932660	AB932695	AB932735	AB932780	AB932825
			*nigromaculata*	Palearctic	Sapporo, Japan	AB932664	AB932699	AB932742	AB932787	AB932832
			*phalerata*	Palearctic	TMU	AB932667	AB932702	AB932746	AB932791	AB932836
			*quinaria*	Nearctic	DSSC (15130–2011.00)	AB932670	AB932705	AB932749	AB932794	AB932839
			*recens*	Nearctic	University of Rochester	AB932671	AB932706	AB932750	AB932795	AB932840
			*subpalustris*	Nearctic	TMU	AB932675	AB932710	AB932755	AB932800	AB932845
			*tenebrosa*	Nearctic	University of Rochester	AB932676	AB932711	AB932756	AB932801	AB932846
			*transversa*	Palearctic, Nearctic	University of Rochester	AB932678	AB932713	AB932758	AB932803	AB932848
			*unispina*	Palearctic, Oriental	Sapporo, Japan	AB932680	AB932715	AB932760	AB932805	AB932850
		*testacea*	*orientacea*	Palearctic	Sapporo, Japan	AB932665	AB932700	AB932743	AB932788	AB932833
			*putrida*	Nearctic	TMU	AB932669	AB932704	AB932748	AB932793	AB932838
			*testacea*	Palearctic, Oriental	Sapporo, Japan	AB932677	AB932712	AB932757	AB932802	AB932847
		*tripunctata*	*tripunctata*	Nearctic, Neotropical	TMU	AB932679	AB932714	AB932759	AB932804	AB932849
	*Siphiodora*	*virilis*	*virilis*	Cosmopolitan	GenBank	AB033640	D10697	JQ679111	EU493791	AF184014
		*repleta*	*hydei*	Cosmopolitan	GenBank	X58694	L41650	JQ679112	EU493736	HQ110530
*Idiomyia*			*grimshawi*	Australasian (Hawaii)	GenBank	CH916372	CH916368	CH916668	CH916668	CH930377

Classification to subgenus and species group, distribution range, source and Genbank accession numbers of mitochondrial and nuclear sequences (underlined for newly determined ones) are given for each species.

### DNA extraction, PCR, cloning and sequencing

Genomic DNA was extracted from individual flies by the method of Stellaer [25] or Boom et al. [26] with some modifications [27]. PCR amplifications were carried out in 10-μl reaction volumes, each containing 1X Ex *Taq* buffer (Takara Bio), 200 μM dNTP, 0.5–1.0 μM of each primer, 0.25 U Ex *Taq* (Takara Bio), and approximately 10 ng of genomic DNA.

PCR thermal cycling conditions were 1 min at 94°C; 35 cycles of 30 s at 94°C, 30 s at annealing temperature and 90 s at 72°C; and 7 min at 72°C. Annealing temperatures were 45°C (*COI*), 49°C (*Adh*), 51°C (*COII*), 55°C (*28S*), or 60°C (*Gpdh*). The primers used for the amplifications are listed in Table 2. Almost all of the PCR products were sequenced directly. However, 27 *Adh* and nine *Gpdh* sequences were sequenced after cloning into pGEM-T Easy vector (Promega) using *E*. *coli* DH5α as the host. Sequences were determined in both directions using a BigDye Terminator Sequencing Kit (Life Technologies) and an ABI 3130 and 3730 Genetic Analyzer, according to the manufacturer’s protocols.

**Table 2:** List of primers for the target genes.

Locus	Length (bp)	Primers
*Adh*	711	Adh-F: 5’-ATGGCAATCGCTAAGAA-3’
		Adh-R:5’-TTAGATGCCAGAGTCCCAGT-3’
*Gpdh*	699	Gpdh-F:5’-GTTTCTAGATCTGGTTGAGGCTGCCAAGAA-3’
		Gpdh-R:5’-ACATATGCTCTAGATGATTGCGTATGCA-3’
*28S*	668	28S-F:5’-GACTACCCCCTGAATTTAAGGAT-3’
		28S-R:5’-CTCCTTGGTGCGTGTTTC-3’
*COI*	699	COI-F:5’-CAACATTTATTTTGATTTTTTGG-3’
		COI-R:5’-TYCATTGCACTAATCTGCCATATTAG-3’
*COII*	666	COII-F:5’-ATGGCAGATTAGTGCAATGG-3’
		COII-R:5’-GTTTAAGAGACCACTTG-3’

### Phylogenetic analyses

Nucleotide sequences were aligned with the MUSCLE [28] algorithm implemented in MEGA 5.05 [29] with the default settings: gap opening = -400, gap extend = 0, max iterations = 8, clustering method = UPGMB and minimum diagonal length = 24. The intron sequences of *Adh* and *Gpdh* were completely removed. Gaps were produced in the alignment procedure. The aligned sequence of *28S* only included 15 gap sites. The resulting alignments were checked by eye. The aligned sequences of the five genes were concatenated by using FASconCAT [30] and the total length of the concatenated alignment was 3,443 bp (S1 Dataset).

We used the maximum-likelihood (ML) and Bayesian inference (BI) methods to construct phylogenetic trees. Partitioning concatenated data into more homogeneous subsets improves parameter estimation, reducing small data subset effects that can reduce signal-to-variance ratios [31]. Therefore, the coding sequences of each gene were partitioned into the 1st+2nd and 3rd codon positions, but the *28S* sequences were not partitioned. The optimal substitution models for ML and BI were selected by using jModelTest 2.2 [32,33] on the basis of the corrected Akaike information criterion (AICc) [34]. Partitions and their associated substitution models are shown in Table 3.

**Table 3:** Partitions and their associated substitution models.

Partition	Model	Base frequencies	Rate matrix	I	G
Adh-12	GTR+G+I	A = 0.3111	A-C = 1.4666	0.4520	0.6850
		C = 0.2243	A-G = 2.7495		
		G = 0.2425	A-T = 0.8934		
		T = 0.2222	C-G = 1.4040		
			C-T = 4.1527		
			G-T = 1.0000		
Adh-3	GTR+G	A = 0.1335	A-C = 2.5655	-	3.3950
		C = 0.3391	A-G = 6.5409		
		G = 0.2597	A-T = 3.4327		
		T = 0.2678	C-G = 0.7696		
			C-T = 5.2957		
			G-T = 1.0000		
Gpdh-12	TrN+I	A = 0.2943	A-C = 1.0000	0.8820	-
		C = 0.1853	A-G = 1.3680		
		G = 0.2758	A-T = 1.0000		
		T = 0.2447	C-G = 1.0000		
			C-T = 15.8711		
			G-T = 1.0000		
Gpdh-3	GTR+G	A = 0.1502	A-C = 1.2973	-	1.6310
		C = 0.3400	A-G = 8.8768		
		G = 0.2256	A-T = 2.1824		
		T = 0.2843	C-G = 0.5332		
			C-T = 5.2132		
			G-T = 1.0000		
28S	HKY+I+G	A = 0.3705	kappa = 4.1913	0.6400	0.3840
		C = 0.1220			
		G = 0.1746			
		T = 0.3329			
COI-12	GTR+I	A = 0.2382	A-C = 4.8670	0.8840	-
		C = 0.2145	A-G = 58.8736		
		G = 0.1955	A-T = 10.2897		
		T = 0.3518	C-G = 5.7327		
			C-T = 141.3394		
			G-T = 1.0000		
COI-3	TrN+I+G	A = 0.3972	A-C = 1.0000	0.0310	0.6690
		C = 0.0688	A-G = 149.3167		
		G = 0.0232	A-T = 1.0000		
		T = 0.5108	C-G = 1.0000		
			C-T = 62.3276		
			G-T = 1.0000		
COII-12	GTR+I	A = 0.2732	A-C = 9.1372	0.8740	-
		C = 0.1767	A-G = 30.9179		
		G = 0.1910	A-T = 4.7341		
		T = 0.3591	C-G = 0.0016		
			C-T = 102.0646		
			G-T = 1.0000		
COII-3	HKY+I	A = 0.4436	kappa = 67.2853	-	0.3700
		C = 0.0652			
		G = 0.0239			
		T = 0.4673			

Models listed here were used in Bayesian analyses.

The ML analysis was performed using RAxML 8.0.26 with RAxMLGUI 1.3.1 [35,36]. To obtain the ML tree, we used the rapid hill-climbing algorithm [37] with the 1,000 slow bootstrap (BP) option. The evolutionary model used for each data partition was GTR+I+G.

MrBayes 3.2.3 [38,39] was used to obtain the BI tree. For BI analyses, a starting tree was randomly selected and four chains were run. In each BI analysis, two independent runs were implemented in parallel, with trees sampled every 1,000 cycles. Runs were stopped after 5,000,000 Markov Chain Monte Carlo (MCMC) cycles. For each run, 5,001 samples were obtained, among which 2,500 early-phase trees were discarded as burn-in. The trace files generated by the MCMC runs were inspected in TRACER 1.6.0 [40] to check whether the number of sampling generations and effective sample sizes (ESS) were large enough for reliable parameter estimates. A consensus of sampled trees was computed using the“sumt” command, and the posterior probability (PP) for each interior branch was obtained to assess the robustness of the inferred relationships.

### Estimation of divergence times

Divergence times were estimated using the relaxed molecular clock method implemented in BEAST 2.1.3 [41]. This method permits evolutionary rate variation among lineages. An uncorrelated lognormal rate evolutionary model was assumed for descendant lineages, and MCMC was used to estimate the parameters. The alignment and evolutionary models of nucleotide substitutions were the same as those used in the phylogenetic tree construction. A Yule prior was applied for the speciation process. Four MCMC chains of 50 million generations each were sampled every 1,000th generation. We collected 75% post burn-in samples and used TRACER to check whether the number of generations and ESS were large enough for reliable parameter estimation. No fossil records that can be used as calibration points exist in the subgenus *Drosophila*, hence we adopted the following estimates as calibration points from Russo et al. [20]: the estimated crown ages for the revised subgenera *Drosophila* (34.5 ± SD 12.3 Mya) and *Siphlodora* (27.1± 4.6 Mya) were introduced as priors with normal distributions in the analysis.

### Biogeographical analysis

Distribution ranges of extant species were characterized by six unit biogeographic areas (OR: Oriental; PA: Palearctic; NA: Nearctic; NT: Neotropical; AU: Australasian; and AF: Afrotropical) and their combinations, referring to DrosWLD [42]. In order to infer possible ranges of ancestral species, the Bayesian Binary MCMC (BBM) method implemented in RASP 3.0 [43] was used. This method is primarily designed for reconstructing the ancestral state of a given node. In the BBM analysis, the probabilities of ancestral ranges at a node were estimated in terms of the MrBayes-generated probabilities of the unit biogeographic areas or their combinations. For estimating the possible ranges of ancestral species, 50,001 binary trees generated by BEAST 2.1.3 were used as input trees, with 12,501 discarded as burn-in. The maximum number of possible ancestral ranges at each node was set to six and the model of BBM analysis was as follows: the state frequency was fixed (Jukes-Canter) and the among-site rate variation was equal. For inferring possible ranges of ancestral species, 10 chains were run simultaneously for 50,000 generations of MCMC. The state was sampled every 100 trees and 100 early-phase trees were discarded as burn-in.

## Results

### Phylogeny

**Fig 1:**
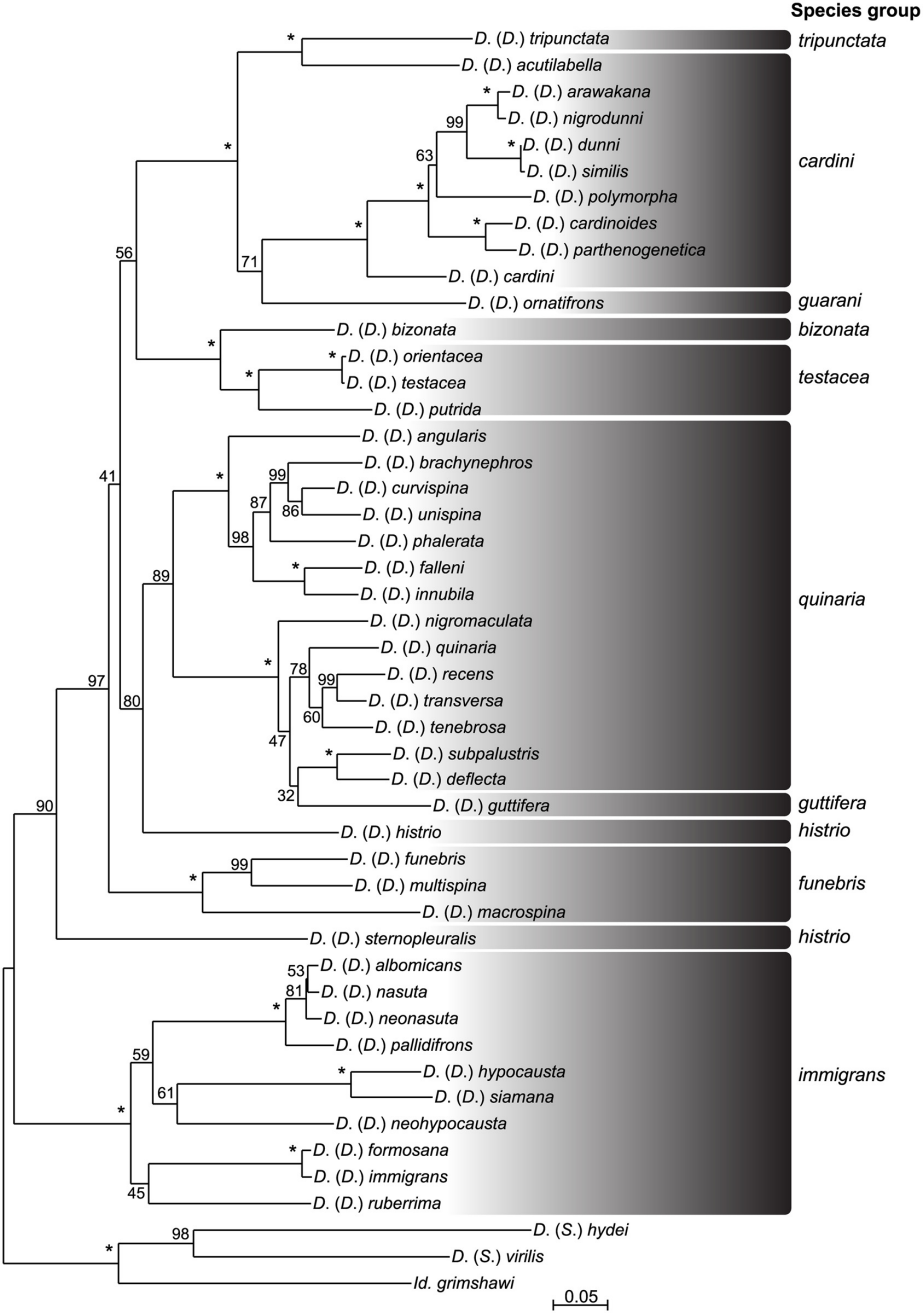
Phylogenetic tree constructed by the Maximm likelihood (ML) analysis of the concatenated dataset. Bootstrap values (BP) inferred by RAxML analysis are shown along interior branches. Symbol “*” indicates 100% BP.

**Fig 2:**
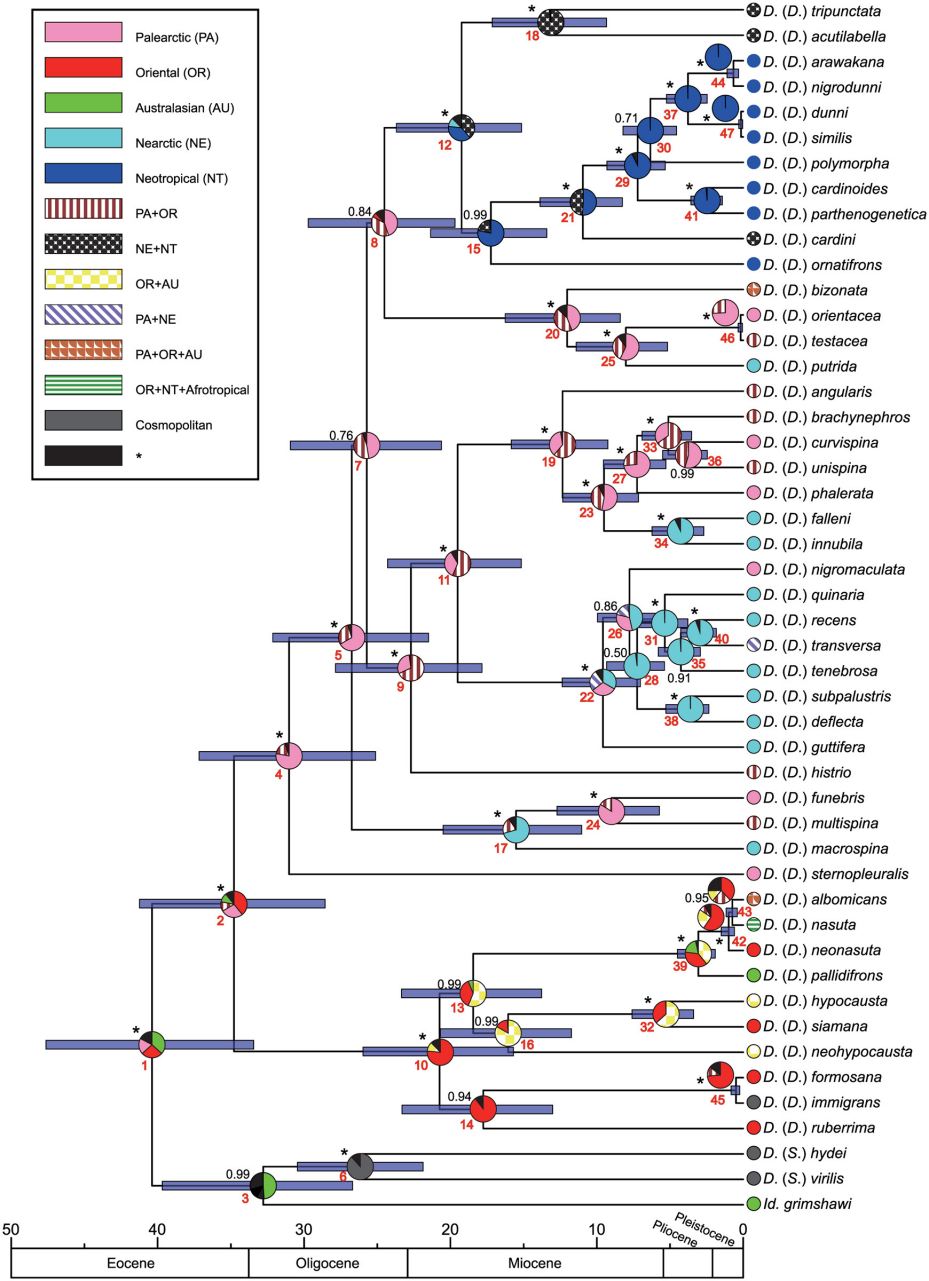
Maximum clade credibility tree showing divergence time and reconstructed ancestral geographical distribution for each extant and ancestral species. Blue bars show 95% time range intervals. Pie charts along nodes indicate probability of ancestral distribution estimated by BBM analysis. Ancestral distribution colored black means any ancestral distributions with relative probabilities <10%. Posterior probability (PP) inferred by BEAST analysis are shown along interior branch. Symbol “*” indicates 100% support value. Extant geographical distributions were retrieved from DrosWLD, shown in Table 1. The red typeface numbers indicate node numbers corresponding to those in Table 4.

Fig 1 shows the best-scoring maximum likelihood (ML) tree obtained from the RaxmlGUI analysis using the concatenated dataset of five genes (*Adh*, *Gpdh*, *28S*, *COI*, and *COII*). BEAST and MrBayes generated similar tree topologies, with only minor differences (Fig 2 and S1 Fig) that did not affect our conclusions.

In Figs 1 and 2, the ingroup species of the subgenus *Drosophila* were divided into two major clades, the *immigrans* species group and a clade that included all the remaining species groups. Within the *immigrans* group, the basal topology was not highly resolved except for the strongly supported monophyly of the *nasuta* species subgroup including *D*. *albomicans*, *D*. *nasuta*, *D*. *neonasuta* and *D*. *pallidifrons*. Although the *immigrans* subgroup (*D*. *immigrans*, *D*. *formosana* and *D*. *ruberrima*) and the *hypocausta* subgroup (*D*. *hypocausta*, *D*. *neohypocausta* and *D*. *siamana*) also formed monophyletic groups, the support for these clades was not as high, especially in the ML tree (Fig 1).

The other clade comprised 35 species belonging to nine species groups (*bizonata*, *cardini*, *funebris*, *guarani*, *guttifera*, *histrio*, *quinaria*, *testacea* and *tripunctata*). Among the other species groups that included multiple representatives, only two, the *funebris* group and the *testacea* group, were recovered as highly supported clades. The *quinaria* group was paraphyletic, but formed a highly supported clade along with the monotypic *guttifera* group. The clade including the *quinaria* + *guttifera* groups was split into two subclades with high confidence; one was comprised of *D*. *angularis*, *D*. *brachynephros*, *D*. *curvispina*, *D*. *unispina*, *D*. *phalerata*, *D*. *falleni* and *D*. *innubila*, and the other of *D*. *quinaria*, *D*. *tenebrosa*, *D*. *deflecta*, *D*. *subpalustris*, *D*. *recens*, *D*. *transversa*, *D*. *nigromaculata* and *D*. *guttifera*. In the latter subclade, the phylogenetic positions of *D*. *guttifera*, *D*. *nigromaculata*, *D*. *quinaria* and *D*. *tenebrosa* were largely different among the analyses (Figs 1 and 2 and S1 Fig). The two species of the *histrio* group were placed distantly: *D*. *sternopleuralis* was placed as the most basal branch to the clade comprising all the remaining 34 species, but *D*. *histrio* was assigned as the sister branch to the *quinaria* + *guttifera* clade. *D*. *bizonata* (the *bizonata* group) formed a highly supported clade with the *testacea* group. The three species groups (*tripunctata*, *cardini* and *guarani*) endemic to the New World formed another highly supported clade. Within this clade, however, the *cardini* group was not monophyletic. One of its members, *D*. *acutilabella*, formed a clade with *D*. *tripunctata* (the *tripunctata* group). However, the confidence for the basal relationships among four lineages (nodes 9, 12, 17 and 20 in Fig 2) was not very high.

### Biogeographic history

Possible ancestral distributions inferred by the BBM analysis and divergence time estimates inferred by the BEAST analysis are shown in Fig 2. The Most Recent Common Ancestor (MRCA) of the subgenus *Drosophila* was estimated to have been distributed in the Oriental (OR) and/or Palearctic (PA) regions (OR: 39%; PA: 27%; PA+OR: 12%; Australasia: 11%) in the late Eocene to early Oligocene around 34.8 Mya (node 2, Table 4). From this ancestor, two lineages diverged in different geographic regions: the MRCA of the *immigrans* group was most likely distributed in OR (76%) at about 20.7 Mya (node 10), while the MRCA of the other lineage including the *bizonata*, *cardini*, *funebris*, *guarani*, *guttifera*, *histrio*, *quinaria*, *testacea* and *tripunctata* groups in PA (78%) was likely distributed at about 31.0 Mya (node 4). The most recent range expansion was seen in *D*. *nasuta* (node 43), which very recently invaded the Neotropical (NT) region from its original range of AF and OR [44].

**Table 4:** Divergence time among nodes, and ancestral distribution at each node estimated by the BBM analysis.

Clade No.	Divergence time (Mya)	95% range	Ancestral distribution (relative probability)
1	40.39	47.63–33.44	AU (0.37), OR (0.27), PA (0.19), * (0.17)
2	34.78	41.24–28.56	OR (0.39), PA (0.27), PA+OR (0.12), AU (0.11), * (0.11)
3	32.79	39.68–26.68	AU (0.49), * (0.51)
4	31.02	37.17–25.11	PA (0.78), PA+OR (0.17), * (0.05)
5	26.74	32.13–21.50	PA (0.67), PA+OR (0.29), * (0.05)
6	26.11	30.45–21.87	Cosmopolitan (0.88), * (0.11)
7	25.71	30.93–20.62	PA (0.48), PA+OR (0.48), * (0.05)
8	24.51	29.71–19.69	PA (0.45), PA+OR (0.38), * (0.07)
9	22.70	27.85–17.84	PA+OR (0.68), PA (0.30), * (0.02)
10	20.74	25.97–15.70	OR (0.76), * (0.12)
11	19.50	24.28–15.16	PA+OR (0.56), PA (0.35), * (0.09)
12	19.24	23.69–15.14	NA+NT (0.39), NT (0.38), NA (0.11), * (0.12)
13	18.44	23.34–13.77	OR+AU (0.55), OR (0.37), * (0.06)
14	17.76	23.3–13.02	OR (0.90), * (0.10)
15	17.24	21.36–13.42	NT (0.78), NA+NT (0.21), * (0.01)
16	16.06	20.69–11.74	OR+AU (0.83), OR (0.16), * (0.01)
17	15.52	20.5–11.04	NA (0.71), PA+OR (0.20), * (0.09)
18	13.14	17.14–9.34	NA+NT (0.98), * (0.02)
19	12.34	15.85–9.25	PA+OR (0.61), PA (0.36), * (0.02)
20	12.02	16.26–8.40	PA (0.44), PA+OR (0.43), * (0.13)
21	10.95	13.88–8.25	NT (0.51), NA+NT (0.48), * (0.01)
22	9.58	12.37–7.02	NA (0.34), PA (0.31), PA+NA (0.26), * (0.10)
23	9.51	12.33–7.15	PA (0.53), PA+OR (0.40), * (0.07)
24	8.99	12.71–5.73	PA (0.84), * (0.16)
25	8.02	11.41–5.17	PA (0.57), PA+OR (0.32), * (0.10)
26	7.77	9.95–5.78	NA (0.46), PA (0.33), * (0.19)
27	7.25	9.55–5.29	PA (0.74), * (0.26)
28	7.25	9.33–5.38	NA (0.98), * (0.02)
29	7.21	9.31–5.32	NT (0.93), * (0.07)
30	6.34	8.21–4.55	NT (1.00)
31	5.36	7.11–3.79	NA (1.00)
32	5.27	7.59–3.39	OR+AU (0.63), OR (0.36), * (0.01)
33	5.12	6.91–3.53	PA+OR (0.65), PA (0.34)
34	4.28	6.22–2.69	NA (0.93), * (0.07)
35	4.26	5.80–2.93	NA (0.99), * (0.01)
36	3.87	5.50–2.46	PA (0.55), PA+OR (0.45)
37	3.77	5.23–2.47	NT (1.00)
38	3.69	5.28–2.35	NA (1.00)
39	3.06	4.49–1.91	OR+AU (0.39), OR (0.38), AU (0.19), * (0.04)
40	2.95	4.27–1.84	NA (0.95), * (0.05)
41	2.41	3.57–1.43	NT (0.99), * (0.01)
42	0.99	1.50–0.60	OR (0.60), OR+AU (0.25), * (0.15)
43	0.74	1.17–0.41	OR (0.38), PA+OR (0.23), OR+AU (0.14), * (0.25)
44	0.66	1.10–0.32	NT (1.00)
45	0.49	0.83–0.24	OR (0.74), PA+OR (0.12), * (0.14)
46	0.17	0.35–0.06	PA (0.74), PA+OR (0.25), * (0.01)
47	0.15	0.30–0.04	NT (1.00)

Asterisks indicates the ancestral areas each of which with the relative probability of < 0.1.

Dispersals from the Old World to the New World were inferred to have occurred five times in the latter lineage. The first migratory event occurred from the PA (45%) or PA+OR (38%) distribution (node 8) to Nearctic (NA)+NT (39%) or NT (38%) (node 12) in the *tripunctata*, *cardini* and *guarani* group clade. The second event was detected in the ancestor of the *funebris* group, which migrated from PA (67%) or PA+OR (29%) to the NA (71%) during the period of 26.7 (node 5) to 15.5 (node 17) Mya. The third event was a migration or range expansion in the ancestor of the Nearctic *quinaria*-group clade including *D*. *guttifera*, which occurred at 19.5 (node 11) to 9.6 (node 22) Mya. The fourth was a migration in the ancestor of *D*. *falleni* and *D*. *innubila* from PA (53%) or PA+OR (40%) to NA (93%) 9.5 (node 23) to 4.3 (node 34) Mya. The fifth was a migration of *D*. *putrida* from PA (57%) or PA+OR (32%) to NA (current range) after 8.0 Mya (node 25).

In addition, dispersals in the reverse direction from the New World to the Old World were inferred in a few individual species and a small clade. From the above-mentioned data, Nearctic MRCA of the *funebris* group, the ancestor of the Old World species *D*. *funebris* and *D*. *multispina*, migrated back to PA (84%) or PA+OR (16%) 26.7 (node 17) to 15.5 (node 24) Mya. Within the Nearctic clade of the *quinaria* group, there are two species that are not endemic to NA: *D*. *nigromaculata* distributed in PA and *D*. *transversa* distributed in the Holarctic (PA+NA). The PA distribution of *D*. *nigromaculata* would have resulted from the backward migration of this species from the NA ancestor or vicariant speciation of the Holarctic ancestor into this PA species and the NA ancestor of the other species. However, this inference was not supported in the ML tree (Fig 1) or BI tree (S1 Fig): *D*. *nigromaculata* was placed basal to the clade containing *D*. *guttifera* and the other six species of the *quinaria* group. *D*. *transversa* expanded its range to PA after 3.0 Mya (node 40).

## Discussion

### Phylogeny

The phylogeny reconstructed by the present study was largely consistent with those in previous studies. The subgenus *Drosophila* (the traditional *immigrans*-*tripunctata* radiation) was consistently divided into two clades: one was the *immigrans* species group and the other comprised the remaining species groups [2,5,13,14,20,23,24]. Although the *immigrans* group has thus far been regarded as monophyletic, this may be due to insufficient taxon-sampling for this species group. Recent studies have revealed that *D*. *quadrilineata*, a member of the *quadrilineata* subgroup of the *immigrans* group, was placed outside the clade of the *immigrans* group proper [4,12,19], and that it is closely related to the genus *Samoaia* [6,45]. Moreover, Katoh et al. [12] suggested that *D*. *annulipes*, another species of the *quadrilineata* subgroup, is related to the genus *Idiomyia* (or Hawaiian *Drosophila*), along with *D*. *maculinotata*. Thus, the *quadrilineata* subgroup is still questionable in terms of its monophyly and phylogenetic position [12]. Another questionable species subgroup in the *immigrans* group is the *curviceps* subgroup. When Zhang and Toda [46] established this species subgroup, they suggested that it is closer to the *quadrilineata* subgroup than to the other subgroups of the *immigrans* group. Yassin [6] did not assign these two species subgroups to any subgenus in his revised classification of *Drosophila*. This taxonomic situation is inconvenient: some members (the *immigrans*, *nasuta* and *hypocausta* subgroups) of the *immigrans* group belong to the subgenus *Drosophila*, but the others (the *quadrilineata* and *curviceps* subgroups) are *incertae sedis* for the subgenus. To partly solve this problem, Pradhan et al. [47] have made the *curviceps* subgroup independent, as a species group, from the *immigrans* group, but left its subgeneric assignment undetermined, i.e., *incertae sedis*. Thus, more taxonomic revision is needed for the traditional *immigrans* group and its related taxa.

The clade including the species groups other than the *immigrans* group is here termed the *quinaria*-*tripunctata* radiation and is characterized by the less resolved, basal branching of major component clades. Within this radiation, two large, well-supported clades were recognized in agreement with previous studies. One is the so-called *tripunctata* radiation, the monophyly of which has been repeatedly inferred in the previous studies [13,14,22–24]. Currently, this radiation includes 123 species of six (*calloptera*, *cardini*, *guarani*, *pallidipennis*, *sticta* and *tripunctata*) species groups that diversified mostly in the Neotropical region [22,24,42]. Phylogenetic relationships among these species groups and the monophyly for some of them are still questionable [5,13,14,23,24], and the present study cannot address these issues because of poor taxon-sampling (only 11 species from three species groups). The other large, well-supported clade comprises the *quinaria* and *guttifera* species groups. It has often been indicated in previous analyses [4,5,13,19–21,48] that *D*. *guttifera* is closely related to the *quinaria* group. Accordingly, Markow and O'Grady [49] treated *D*. *guttifera* as a species of the *quinaria* group. The present study has confirmed the inference by Perlman et al. [21] that this “*quinaria*” clade is further divided into two subclades. These subclades can be more or less distinguished from each other by their choice of breeding niches: one consists of fungal breeders such as *D*. *phalerata*, *D*. *brachynephros* and *D*. *falleni*, whereas the other includes decaying plant breeders such as *D*. *nigromaculata*, *D*. *deflecta*, *D*. *quinaria*, *D*. *limbata*, D. *subpalustris* and *D*. *palustris* [21,50,51].

However, some phylogenetic inferences drawn from the present study are new or different from those of previous studies. The phylogeny of the *histrio* species group has been unclear for a long time, although either *D*. *histrio* or *D*. *sternopleuralis* has been included in some previous studies [3–6,20,21]. The present study included both species and revealed that the *histrio* group is not monophyletic. With high confidence, *D*. *sternopleuralis* represents the most basal lineage in the *quinaria*-*tripunctata* radiation, while *D*. *histrio* is the sister taxon to the “*quinaria*” clade. The phylogenetic position of *D*. *sternopleuralis* has been problematic in previous studies [3,4,20]. It should be noted, however, that Russo et al. [20] placed *D*. *sternopleuralis* along with *D*. *pruinosa* of the Afrotropical *loiciana* species complex [52] in the sister clade to the *immigrans* group proper, though not highly supported. Furthermore, although *D*. *sternopleuralis* is distributed in warm temperate regions of Japan and Korea, a number of its relatives have been recorded in the tropical Australasian to the subtropical Oriental regions, i.e., Papua New Guinea, Borneo, Sri Lanka, India, Nepal, Myanmar, southern China and Taiwan [43,53–56]. Although the phylogenetic position of *D*. *histrio* has also been controversial [4–6,21], our results strongly support that it is a sister relationship to the “*quinaria*” clade, which is consistent with the topology inferred by van der Linde and Houle [4].

Only two previous studies [5,6] examined the monophyly of the *funebris* species group, including *D*. *funebris* and *D*. *macrospina*, in molecular phylogenetic analyses. However, the results were controversial. The present study suggests that three species, *D*. *multispina* in addition to the two above, of the *funebris* group form a highly supported clade. Although its placement within the *quinaria*-*tripunctata* radiation is consistent with most previous studies [2,3–6,9,14,19–22], its phylogenetic position within the radiation has been uncertain in previous studies.

The *testacea* and *bizonata* species groups formed a clade in previous, phylogenetic studies [5,6], and our analyses confirm this, but differ from that of van der Linde et al. [5] with respect to the phylogenetic position of *D*. *bizonata*. We placed it as the sister to the *testacea* group, which is monophyletic. However, van der Linde et al. [5] placed *D*. *bizonata* within the *testacea* group, rendering the latter paraphyletic. The discrepancy may have been due to the difference in gene markers employed for the phylogenetic analyses. In addition, both studies included only one species (*D*. *bizonata*) of the *bizonata* group, of which six more species are known. This issue should be addressed using better taxon-sampling.

### Evolutionary history

Throckmorton [1] hypothesized that two major biogeographic patterns resulted in the evolution of the Drosophilidae. The first is the primary disjunction of tropical faunas between the Old and the New Worlds in the early Oligocene, and the second is the disjunction of temperate faunas between the two Worlds in the mid-Miocene. A number of recent studies have tested this hypothesis by molecular phylogenetic analyses in specific lineages [17,18,24] or across large taxa such as the genus *Drosophila* [19], the subfamily Drosophilinae [23] and the entire family Drosophilidae [20]. However, the time estimates for these disjunctions varied among the studies because of differences in the timescale placed on the phylogenies that were used [57]. Dates from two sources have been used as calibration points to estimate divergence times within the Drosophilidae: one from fossils, and the other from biogeography. The former source is rare in Drosophilidae, with only few Baltic and Dominican amber fossils dated geologically [58,59]. The latter source comes from the phylogeography of Hawaiian drosophilids, and has more often been used to establish calibration points in molecular phylogenetic studies on Drosophilidae [60,61]. However, Obbard et al. [57] pointed out some biases of the Hawaiian-calibrated dates. Secondarily, taxon sampling affects the inferences on evolutionary history through estimations of ancestral distributions and divergence times. Gao et al. [18] pointed out the importance of recognizing genuine sister groups in obtaining reliable estimates for these attributes.

Since neither fossil-based nor phylogeography-based calibration points are available for the species employed in the present study, we applied the divergence time estimates at two nodes (node 2 and node 6) from Russo et al. [20] as calibration points. The study by Russo et al. employed the largest number of calibration points (two fossil-based and six Hawaiian-phylogeographic calibrations) for estimating divergence times, and was based on the largest taxon samplings (358 species) from the Drosophilidae, but it was somewhat biased to New World species for the *quinaria*-*tripunctata* radiation. The present study was conducted to compensate for this bias by including more Old World species of this radiation.

Russo et al. [20] inferred that the pattern of drosophilid faunal disjunctions between the Old and the New Worlds was not as simple as Throckmorton [1] depicted. Such disjunctions were regarded as having occurred multiple times during the long geological period from the late Eocene to the Holocene. However, the most important aspect of Throckmorton’s [1] hypothesis, i.e., the parallelism of disjunctions on separate lineages of Drosophilidae, was recovered, indicating that such disjunctions may have been caused by vicariant events. The present study, focusing on the revised subgenus *Drosophila* (the *immigrans*-*tripunctata* radiation), has corroborated the general pattern of disjunctions inferred by Russo et al. [20], but has shown that the evolutionary history of this lineage should largely be revised.

The first difference was seen in the estimation of ancestral distribution for the *quinaria*-*tripunctata* radiation. Russo et al. [20] estimated that the MRCA of this radiation was most likely distributed in the New World, while our results suggested that it was distributed in the Palearctic region. Certainly, this could be due to the difference in taxon sampling between the two studies. Accordingly, Russo et al. [20] inferred that the first Old World–New World disjunction within the subgenus *Drosophila* occurred in the late Eocene to produce two tropical lineages, the *immigrans* species group in the Oriental region and the *tripunctata* radiation in the Neotropical region. In parallel with this disjunction, another vicariant divergence of tropical/subtropical elements was estimated to have concurred in the late Eocene between the Oriental/Palearctic genus *Lordiphosa* and the Neotropical *saltans* and *willistoni* species groups of the subgenus *Sophophora*. However, our results suggest that the ancestor of the *quinaria*-*tripunctata* radiation had once entered the temperate Palearctic region in the early to mid-Oligocene. From this ancestor, then, two lineages, the *funebris* species group and the *tripunctata* radiation, arose by nearly concurrent migrations of their ancestors from the Palearctic region to the New World in the late Oligocene to the early Miocene. Gao et al. [18] estimated that the primary, tropical disjunctions such as that between *Lordiphosa* and Neotropical *Sophophora* occurred in the mid-Eocene when the North Atlantic Land Bridge (NALB) broke. Even if the NALB could serve as a migrating route for organisms such as drosophilid flies with larger dispersal abilities until later ages [20,62], our estimate of the ancestral age of the Neotropical *tripunctata* radiation was much younger than the time of the NALB break. In addition, this lineage was regarded as derived from an ancestor distributed in the Palearctic and/or the Oriental regions in the late Oligocene, when tropical forests continuously graded into temperate forests only in East Asia [62,63]. This suggests that the ancestor of the *tripunctata* radiation, along with the ancestor of the *funebris* group, migrated through the Bering Land Bridge into the Nearctic region. Robe et al. [24] inferred, based on molecular phylogenetic and biogeographic analyses, that after the colonization to the Nearctic region this lineage had expanded its range to the Neotropical region and much diversified there.

The second difference was seen in the estimated time and direction of ancestral migration between the Old and the New Worlds in the “*quinaria*” clade. Russo et al. [20] estimated that the ancestor of this clade had migrated from the New World to the Old World in the late Oligocene. Our divergence time estimates (Fig 2) suggest that it is likely that the ancestor was distributed in the Oriental and/or the Palearctic regions. From this Old World ancestor, one lineage was derived by migration or range expansion to the Nearctic region in the mid-Miocene. Another species concurrently migrated, but in the opposite direction, from the Nearctic to the Palearctic region and became the ancestor of Old World species in the *funebris* group. The Old World–New World temperate disjunctions dated to the mid-Miocene age were detected in the lineages of the genus *Hirtodrosophila* and the *Drosophila* (*Siphlodora*) *melanica* species group as well [20].

The third phase of Old World-New World disjunctions was estimated to have occurred on three lineages in the “*quinaria*” clade and the *testacea* species group, as well as in the *Drosophila* (*Sophophora*) *obscura* species group [20], in the late Miocene. In the fourth phase, the subarctic species *D*. *transversa* achieved its Holarctic distribution by expanding its range from the Nearctic to the Palearctic region in the Pliocene. In this phase, cool-temperate species of the *Drosophila* (*Siphlodora*) *robusta* and *virilis* species groups became disjunct between the Palearctic and the Nearctic regions [20].

Thus, ancestral migrations and subsequent vicariant divergences of descendants between the Old and the New Worlds occurred multiple times in parallel among different lineages in the *quinaria*-*tripunctata* radiation of the subgenus *Drosophila*. Such faunal disjunctions between the two Worlds concurred in other drosophilid lineages as well, and shaped a part of the overall phylogeographic pattern in the family Drosophilidae [20]. These faunal vicariant events correspond to the secondary disjunctions of temperate elements in Throckmorton’s [1] hypothesis, but would have occurred repeatedly in a wider time range, from the late Oligocene to the Pliocene, than Throckmorton [1] proposed. Such multiple migration episodes between the Old and the New World have also been well documented in terrestrial plants and mammals. Tiffney [64] proposed that floristic exchanges between the two Worlds most likely occurred in five major periods: (1) the pre-Tertiary, (2) the early Eocene, (3) the late Eocene–Oligocene, (4) the Miocene, and (5) the late Tertiary–Quaternary periods. The four phases of Old World-New World disjunctions detected for the *quinaria*-*tripunctata* radiation in the present study seem to correspond to the last two of the five flora-exchange periods. Zhanxiang [65] recognized three major waves of dispersals from Eurasia to North America in carnivoran mammals: the first in the early Miocene, the second in the late Miocene and the third in the Pliocene: corresponding to the first, third and fourth phases of faunal disjunctions in the *quinaria*-*tripunctata* radiation, respectively. These parallel, spatiotemporal patterns observed in terrestrial organisms of the northern hemisphere strongly suggest the contribution of the Bering Land Bridge as a common factor to exchanges and isolation of temperate elements between the Old and the New Worlds [20,62,65]. Paleo-climatic and -vegetational conditions around Beringia gradually changed with global cooling after the Early Eocene Climatic Optimum [66]. The vegetation around the Beringia was dominated by rich deciduous and some coniferous temperate forests in the late Oligocene to the early Miocene, but changed to the cool-temperate forest with decreased diversity of deciduous angiosperms and conifers thereafter from the late Miocene to the Pliocene and to the taiga/tundra in the Holocene [62,63]. The changing climatic and vegetational conditions around the Beringia should have filtered different elements of the temperate biota in the process of faunal/floral exchanges between the Old and the New Worlds after the early or mid-Miocene age: elements adapted to the warmer climate became disjunct earlier in geological time.

Our results clearly show the importance of wider and denser taxon sampling for the reconstruction of evolutionary histories, although there still remain a number of species groups of the subgenus *Drosophila* to be included in phylogenetic analyses. Further expansion of taxon sampling across the whole family Drosophilidae should provide more evidence for parallel biogeographic disjunctions in separate lineages, and will help to document the vicariant nature of regional faunal development.

## Supporting Information

S1 Dataset The nexus format file of sequences used in the analyses. (NEX)

S1 Fig Phylogenetic tree constructed by the Bayesian Binary MCMC analysis of the concatenated dataset. (PDF)
