# Supplementary material for: Phylogeography of the Subgenus Drosophila (Diptera: Drosophilidae): Evolutionary History of Faunal Divergence between the Old and the New Worlds
Source: PLoS One. 2016 Jul 27;11(7):e0160051. doi: 10.1371/journal.pone.0160051 (PMC4962979; doi:10.1371/journal.pone.0160051)
Supplement: S1 Fig — (PDF) [file pone.0160051.s002.pdf]

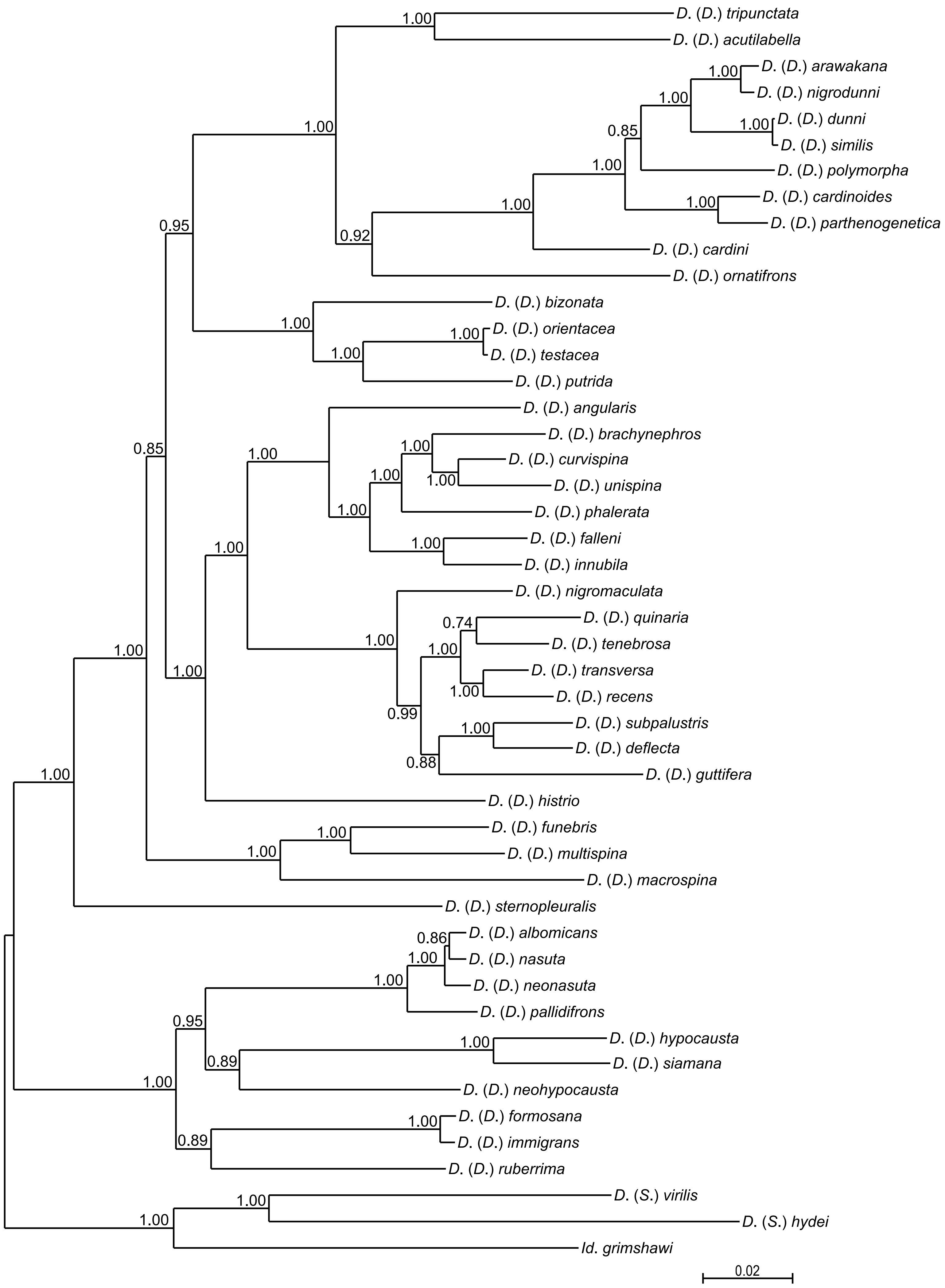

S1 Fig. Phylogenetic tree constructed by the Bayesian Binary MCMC analysis of the concatenated dataset.
